# Greater levels of cardiorespiratory and muscular fitness are associated with low stress and high mental resources in normal but not overweight men

**DOI:** 10.1186/s12889-016-3470-6

**Published:** 2016-08-15

**Authors:** O. Kettunen, H. Kyröläinen, M. Santtila, T. Vuorimaa, T. J. Vasankari

**Affiliations:** 1 Department of Health and Exercise & Paavo Nurmi Center, University of Turku, Turku, Finland; 2 Department of Health and Exercise, Sports Institute of Finland, Vierumäki, Finland; 3 Department of Biology of Physical Activity, University of Jyväskylä, Jyväskylä, Finland; 4 National Defence University, Helsinki, Finland; 5 Haaga-Helia University of Applied Sciences, Vierumäki, Finland; 6 UKK Institute for Health Promotion Research, Tampere, Finland

**Keywords:** Physical fitness, Leisure-time physical activity, Stress, Mental resources, Body weight

## Abstract

**Background:**

The aim of the present study was to investigate how cardio respiratory (CRF) and muscular fitness (MF) together with leisure-time physical activity (LTPA) influence stress symptoms and mental resources among normal-weight and overweight men, because it is not known how body weight affects this association.

**Methods:**

In a cross-sectional study, 824 men (mean ± SD: age 25 ± 5 y, weight 81 ± 13 kg, BMI 25 ± 4 kg/m^2^) underwent CRF and MF tests and completed LTPA and stress questionnaires. For the analysis, the subjects were divided into BMI groups (normal vs. overweight) and CRF / MF / LTPA (low, moderate, high) tertiles.

**Results:**

Normal-weight men with low CRF reported 12 % (*p* = 0.001) more stress symptoms (SS) compared to normal-weight men with moderate CRF, and 13 % (*p* = 0.004) more SS compared to normal-weight men with high CRF. Normal-weight men with low MF reported 13 % (*p* = 0.001) higher SS compared to normal-weight men with moderate MF and 16 % (*p* = 0.002) more SS compared to men with high MF. Among overweight men, there were no significant differences in SS or mental resources (MR) between the low, moderate and high CRF and MF tertiles. Overweight men with high CRF experienced 8 % (*p* = 0.039) more SS compared to normal-weight participants with high CRF when age, tobacco and alcohol use, MF and LTPA were considered as covariates (*p* = 0.014).

**Conclusion:**

Higher CRF and MF are associated with lower stress and higher mental resources in normal-weight men, but in overweight men, these relationships may differ.

## Background

Stress is defined as a nonspecific response of the body to numerous physical, psychosocial and environmental challenges [1, 2]. A growing body of evidence has linked with adverse health effects suggesting that stress should not be overlooked when designing health prevention programs [2]. The role of stress in the etiology of obesity and metabolic syndrome is currently gaining interest [2]. Stress is thought to influence human eating behaviour in two ways, causing either under- or overeating, and chronic life stress may be causally linked to weight gain especially among men [3].

Overweight and obesity are major public health problems worldwide [4, 5]. Overweight is an independent risk factor for a variety of chronic diseases such as diabetes, hypertension, coronary heart disease [6] and depression [7], and it is also associated with high health care costs [6]. Reduced physical health as well as stigmatization associated with overweight can contribute to impaired mental wellbeing among overweight adults [8, 9]. However, opposite opinions exist and the scientific literature has acknowledged the “jolly fat” hypothesis, which predicts that overweight people will have a lower risk of depression and exhibit a lower number of depressive symptoms relative to their normal-weight counterparts [10–13]. Whether this hypothesis applies to stress symptoms is not known.

Physical activity (PA) and cardiorespiratory fitness (CRF) seem to protect against chronic diseases, such as metabolic syndrome [14, 15], type 2 diabetes [16] and cardiovascular disease (CVD) [17, 18]. There is a general belief that PA and exercise have positive effects on mood state and psychological wellbeing, including increased capacity to cope with stress [19], anxiety reduction [20, 21] and mood enhancement [22, 23]. According to some evidence, exercise can prevent the development of stress-related mood disorders, such as depression and anxiety [17]. However, the latest review of the literature investigating the influence of stress on indicators of PA and exercise suggests that it would be wise to acknowledge the bidirectional relationship between stress and PA [24]. The relationship between PA and mental health is likely to be complex and physical inactivity may be the cause and/or the consequence of poor mental health [25].

Low leisure-time physical activity (LTPA) is considered one of the connecting factors between overweight and poor physical fitness (PF) [26, 27]. The aim of the study was to investigate the mutual effect of PA, PF and body composition on stress and mental resources among young men. We hypothesized that the association between mental health variables (stress symptoms and mental resources) and physical fitness/LTPA could be different in normal-weight and overweight young men.

## Methods

### Subjects

The participants in the study were normal, healthy civilian young men who had participated in military services. Because of the compulsory military service, the study population is a geographically representative sample of healthy Finnish civilian young men. The study group was enrolled during eight military refresher courses held around Finland during year 2008. Of the 1155 invited reservists, 922 participated in the courses and 829 (72 % of invited reservists) volunteered for the present study. The participants signed an informed consent form indicating that they were aware of the risks and benefits of the study. The mean (SD) age of the participants was 25 (±5) years, height 180 (±6) cm, weight 81 (±13) kg and body mass index (BMI) 25 (±3.8) kg/m^2^. Thirty-eight percent of the participants were smokers (Table 1).

**Table 1:** Subjects (*n* = 824) characteristics

Character	Mean (±SD)
Age (years)	25 (4)
Height (cm)	180 (6)
Weight (kg)	80 (13)
Body Mass Index (kg/cm^2^)	25 (4)
VO_2_max (ml · kg^-1^ · min^-1^)	40 (8)
Muscle fitness	12 (9)

### Study design

The reservists were informed about the study in the invitation letter for the refresher course.

During the eight refresher courses, all of the measurements were made. In the beginning of each course, a health examination and a discussion about the measurements were carried out. The subjects filled in the questionnaires including health, LTPA and stress questions and underwent cardiorespiratory fitness (CRF) tests and muscular fitness (MF) tests. The weight and height, as well as waist circumference, were measured in the morning after a 12-hour fast. After a light breakfast, muscular fitness was measured using four consecutive tests: grip strength, push-ups, sit-ups, and repeated squats, followed by a cardiovascular fitness test. The ethical committees of the University of Jyväskylä and the Central Finland Health Care District, as well as the Headquarters of the Finnish Defense Forces, approved the study. The detailed study protocol has been reported earlier [28].

### Cardiorespiratory fitness

Oxygen uptake (VO_2_ max) was measured indirectly using a bicycle ergometer test (Ergoline 800 S, Ergoselect 100 K or 200 K, Bitz Germany). The handlebars and seats were individually adjusted. After a 5-minute warm up, the test began with a power output of 75 W, which was increased by 25 W after every other minute. A pedaling rate of 60 rpm was maintained throughout the test. Heart rate (HR) was recorded continuously (Polar Vantage NV or S610, S710 or 810, Kempele, Finland). The test was terminated at volitional exhaustion, including a decrease in the pedaling rate to below 50 rpm. Predicted VO_2_ max was determined from the HR and power (Fitware, Mikkeli, Finland) as follows: VO_2_ max (ml⋅¯^1^kg⋅¯^1^) = 12.35*Pmax/kg + 3.5, where P_max_ is highest work rate (power) achieved during the test as watts and body mass as kilograms. The intraclass correlation has been reported to be high with this method (ICC = 0.82–0.94) for men [29].

### Muscular fitness

For muscular fitness, a muscular fitness index (MFI) was calculated using the result of each muscle test according to the standards of the Finnish Defense Forces [30]. The order of the tests was 1) grip strength (antebrachial muscles) 2) push-ups (arm and shoulder extensors, pectoralis major and triceps brachii), 3) sit-ups (abdominal muscles and hip flexors) and, 4) repeated squats (glutei and quadriceps femoris muscles). Both cardiorespiratory and muscular fitness tests have age-specific reference values used in the Finnish Defense Forces since 2000, and they are based on data of 3635 civilians [30].

The result of the push-ups, sit-ups, and repeated squats were expressed as the number of correctly performed repetitions within 60 s, while the grip strength was measured during a single maximal isometric contraction. Grip strength was determined three times from both hands and the final score was the average of the highest scores of both hands (sitting, elbow at 90°, Saehan Corporation, Masan, South Korea) [31, 32]. In the start position of the sit-up test, the subject was lying supine on the floor with knees flexed at 90° and hands behind his neck. The ankles were fixed to the floor by an assistant, and a repetition was counted after the participant’s elbows touched the flexed knees [33]. When executing one repetition of push-ups, the participant had a shoulder-wide stance and fingers pointing forward. From this start position, the elbows were flexed at 90° with the torso touching the floor. Then the upper extremities were fully extended, while the upper body was straight and fully extended [34]. The repeated squat movement started while standing straight and lowering the upper body until the thighs were at a horizontal level. After this, the subject flexed his lower extremities in order to stand straight again [33]. Before the tests, the supervisors demonstrated the correct technique for each test and, thereafter, they controlled each performance [28].

### Leisure-time physical activity

The weekly LTPA frequency and intensity was determined from responses to a single question (SIVAQ) with six categories: (1) no physical activity at all, (2) some physical activity without feeling out of breath or sweating, (3) physical activity without feeling out of breath or sweating, (4) physical activity without feeling out of breath or sweating twice a week, (5) physical activity without feeling out of breath or sweating three times a week, and (6) physical activity without feeling out of breath of sweating at least four times a week [35]. In the analysis, the subjects were divided into three groups according to their physical activity level: low (LTPA categories 1 and 2 combined), moderate (categories 3 and 4), or high (categories 5 and 6).

The body weight (kg) and height (cm) of the subjects were measured in lightweight clothing. Body mass index (BMI) was calculated for analyses. BMI is a person’s weight in kilograms (kg) divided by his height in meters squared. BMI cut-off points for normal (18.5–24.9) (*n* = 486), overweight (25.0–29.9) (*n* = 264) and obese (≥30.0) (*n* = 74) groups were selected according to the WHO standards [36]. For the analysis, the overweight and obese groups were combined and the study group was divided into normal-weight (BMI < 24.9) (*n* = 486) and overweight groups (BMI ≥ 25) (*n* = 338). The subjects were further divided into six groups according to their BMI (normal vs. overweight) and tertiles of CRF, MF and LTPA (low, moderate, high). Participants were divided into three tertiles of cardiorespiratory fitness according to their VO_2_ max: 1) low CRF <37.87 ml (kg/min, 2) moderate CRF = 37.87–44.87 ml/kg/min, 3) high CRF > 44.87 ml/kg/min; and into three muscle fitness tertiles according to their muscle fitness: 1) low MF < 10.5, 2) moderate MF = 10.5–14.75, 3) high MF > 14.75. Alcohol and tobacco product use were determined using a questionnaire [28].

### Stress

Stress symptoms (SS) were measured using questions from the Occupational Stress Questionnaire (OSQ) [37]. The stress level was calculated using sum scales from questions measuring stress and satisfaction with work and life: A. Stress is defined as the situation when a person feels tense, restless, nervous, or anxious, or is unable to sleep at night because his mind is troubled all the time. Do you feel that kind of stress these days? (1) not at all, (2) only a little, (3) to some extent, (4) rather much, or (5) very much; B. What is your health state compared to that of other people your age? (1) very good, (2) rather good, (3) average, (4) rather poor, or (5) very poor; C. How satisfied are you with your present work? (1) very satisfied, (2) rather satisfied, (3) neither satisfied nor dissatisfied, (4) rather dissatisfied, or (5) very dissatisfied; D. How satisfied are you with your present life? (1) very satisfied, (2) rather satisfied, (3) neither satisfied nor dissatisfied, (4) rather dissatisfied, or (5) very dissatisfied [37]. A high sum meant high stress levels and a low sum meant low stress levels (range of scores 5–20). The validity of the stress questions in the OSQ has been studied, and an association has been identified between psychological symptoms and mental resources. The stress-symptoms item correlated strongly with the mental health scale of the Short-form 36-item Health Survey (–0.63), the content of which emphasizes depressive symptoms, and with the vitality scale (–0.58), which includes items reflecting general energy [38].

### Mental resources

Mental resources (MR) were calculated using a sum scale from three questions with five categories: A. Have you been active and energetic lately? (1) constantly, (2) rather often, (3) now and then, (4) rather seldom, or (5) not at all; B. Do you feel that you are capable and confident? (1) constantly, (2) rather often, (3) now and then, (4) rather seldom, or (5) never; C. Do you think you have done your daily chores well lately? (1) constantly, (2) rather often, (3) now and then, (4) rather seldom, or (5) not at all [37]. A low sum meant high mental resources and high sum meant low mental resources (range of score 3–15).

### Statistics

SPSS software version 18.0 was used for the statistical analysis. The test of normality was conducted using the Kolmogorov-Smirnov test. Means and standard deviations were used for the results and calculated with standard procedures. Means of the stress and mental resources index in the BMI groups were examined with analysis of variance (ANOVA), followed by Bonferroni’s correction as a *post-hoc* test wherein age, alcohol and tobacco consumption, and the other two components of fitness and activity (CRF, MF and LTPA) (ANCOVA) were taken as covariates. Further, the subjects were divided into tertiles based on BMI (normal vs. overweight) and CRF/MF/LTPA (low, moderate, high). Pearson’s correlations, effect sizes (ES) and p for trend were observed as well.

## Results

A weak positive correlation was found to exist between CRF and MF, while a negative correlation was found between CRF, age, BMI, stress and MR (Table 2).

**Table 2:** Correlations between age, Body Mass Index (BMI), cardiorespiratory fitness (CRF) (ml/kg/min), muscular fitness (MF), stress and mental resources (MR)

	BMI	CRF	MF	Stress	MR
Age	.178**	−.105**	.124**	.123**	−.185**
BMI		−.527**	−.220**	−.020	−.026
CRF			.490**	−.166**	−.102**
MF				−.165**	−.142**
Stress					.647

**p < 0.01

There were weak positive correlations between MF and age (*r* = .12, *p* < 0.01), MF and CRF (*r* = .49, *p* < 0.01), as well as weak negative correlations between MF and BMI (*r* = −.22, *p* < 0.01), MF and stress (*r* = −.17, *p* < 0.01), and MF and MR (*r* = −.14, *p* < 0.01) (Table 2).

### Stress and mental resources according to BMI and CRF

**Fig. 1:**
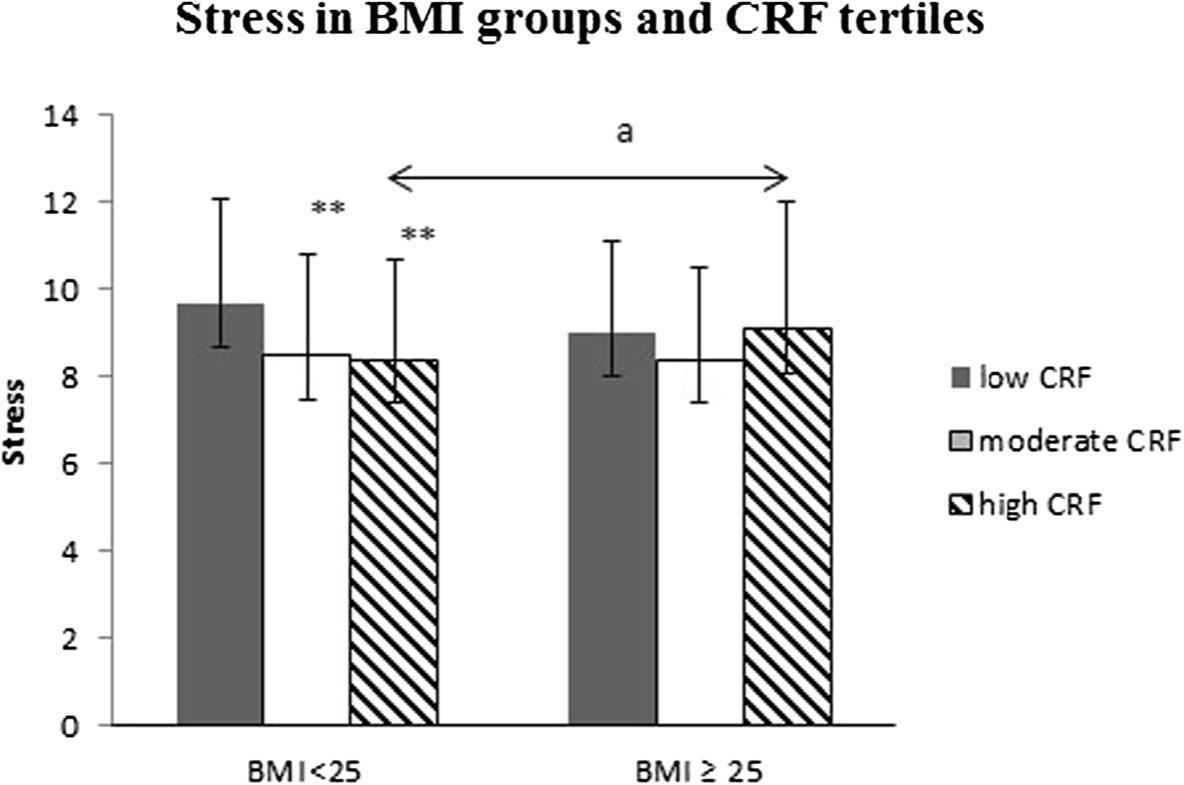
Stress symptoms according to Body Mass Index (BMI) groups and cardiorespiratory fitness (CRF) tertiles. Mean (±SD). The ANCOVA model includes age, tobacco and alcohol use and muscle fitness (MF) and leisure-time physical activity (LTPA) as covariates. Differences between the low and moderate/high subgroups within the BMI < 25 group: *0.01 < *p* ≤ 0.05, **0.001 < *p* ≤ 0.01, ***p < 0.001. Differences between the overweight and normal-weight subgroups within the fitness tertiles: a) *p* ≤ 0.05

There were weak positive correlations between stress and age (*r* = .12, *p* < 0.01), as well as weak negative correlations between stress and CRF (*r* = −.17, *p* < 0.01), stress and MF (*r* = −.17, *p* < 0.01), Normal-weight men with low CRF reported 12 % (ES = 1.20, *p* = 0.001) more stress symptoms compared to the normal-weight men with moderate CRF and 13 % (ES = 1.30 *p* = 0.004) more stress symptoms compared to the normal-weight men with high CRF (Fig. 1). P for trend in stress symptoms among normal weight men was (*p* < 0.001).

There were no significant differences in stress symptoms among overweight men based on CRF tertiles (low/moderate/high). P for trend in stress symptoms among overweight men was (*p* = 0.74).

Overweight men with low CRF (*n* = 160) reported 6 % (ES = 1.07, *p* = 0.055) lower stress symptoms compared to their normal-weight counterparts with low CRF (*n* = 94). No significant differences in stress symptoms existed between the overweight (*n* = 110) and normal-weight (*n* = 155) men with moderate CRF. The overweight subjects with high CRF (*n* = 44) experienced 8 % (ES = 0.61, *p* = 0.039) more stress compared to their normal-weight (*n* = 211) counterparts (Fig. 1).

**Fig. 2:**
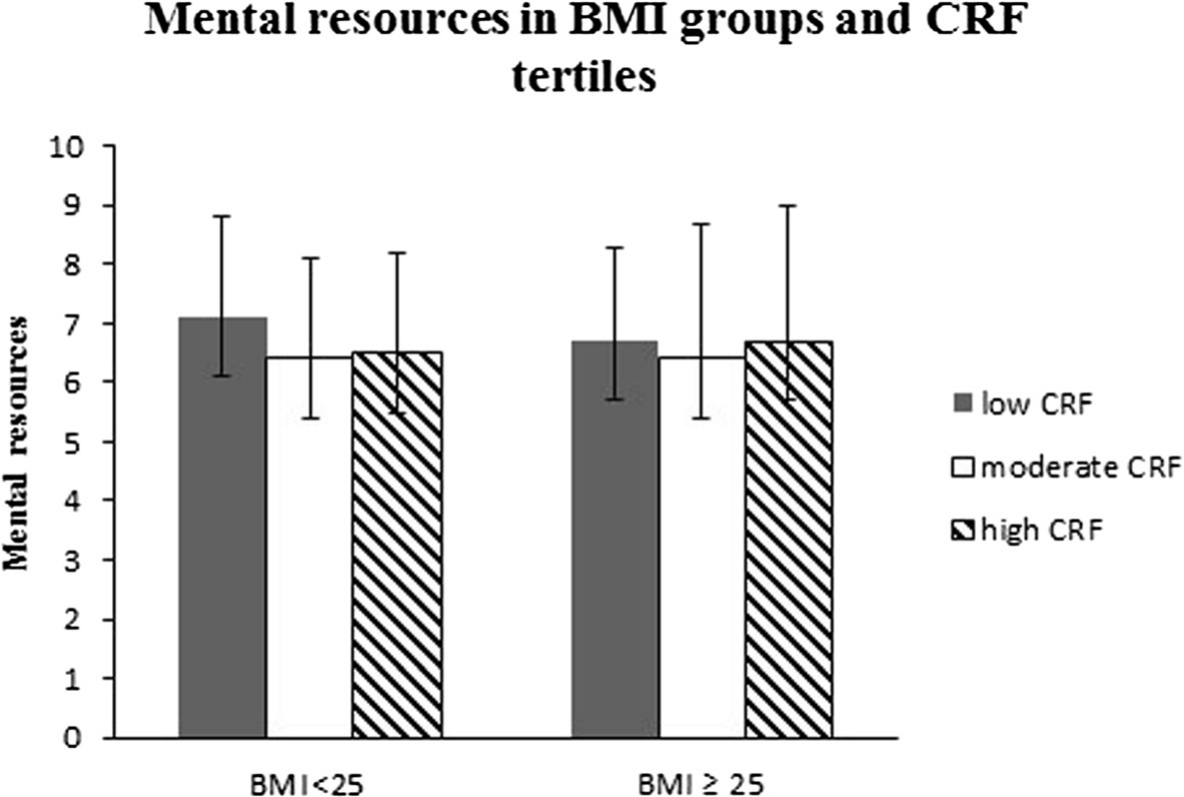
Mental resources according to Body Mass Index (BMI) groups and cardiorespiratory fitness (CRF) tertiles. Mean (±SD). The ANCOVA model contains age, tobacco and alcohol use and muscle fitness (MF) and leisure-time physical activity (LTPA)

There were no significant differences in mental resources between normal-weight and overweight men based on CRF tertiles (*p* = 0.34) or after adjusting with age, alcohol, tobacco, MF and LTPA (*p* = 0.13) (Fig. 2). P for trend in mental resources among normal weight/overweight men was (*p* = 0.001/*p* = 0.43).

### Stress and mental resources according to BMI and MF

Normal-weight men with low MF reported 13 % (ES = 1.27, *p* = 0.001) higher stress symptoms compared to normal-weight men with moderate MF and 16 % (ES = 1.51, *p* = 0.002) more stress symptoms compared to normal-weight men with high MF. P for trend among normal weight men was (*p* < 0.001).

**Fig. 3:**
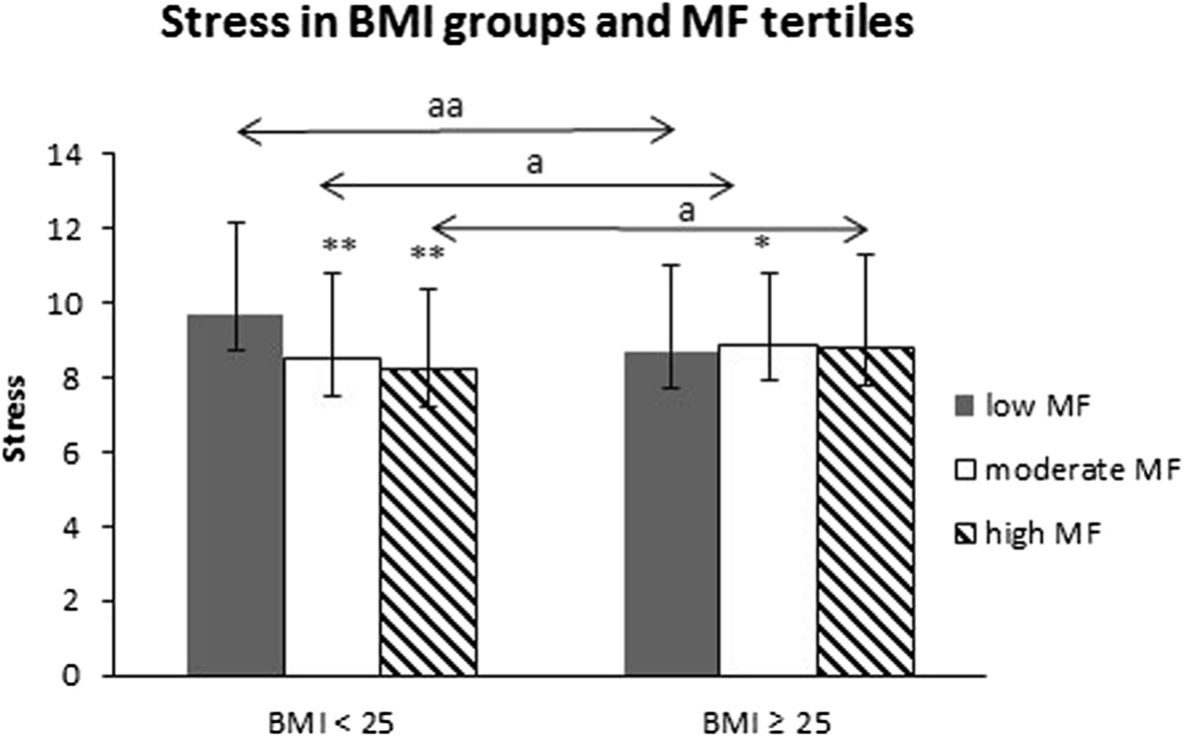
Stress symptoms according to Body Mass Index (BMI) groups and muscular fitness (MF) tertiles. Mean (±SD). The ANCOVA model contains age, tobacco and alcohol use, cardiorespiratory fitness (CRF) and leisure-time physical activity (LTPA) as covariates. Differences between the low and moderate/high subgroups within the BMI < 25 group: *0.01 < *p* ≤ 0.05, **0.001 < *p* ≤ 0.01, ***p < 0.001. Differences between the overweight and normal-weight subgroups within the fitness tertiles: a) *p* ≤ 0.05; aa) *p* < 0.01

**Fig. 4:**
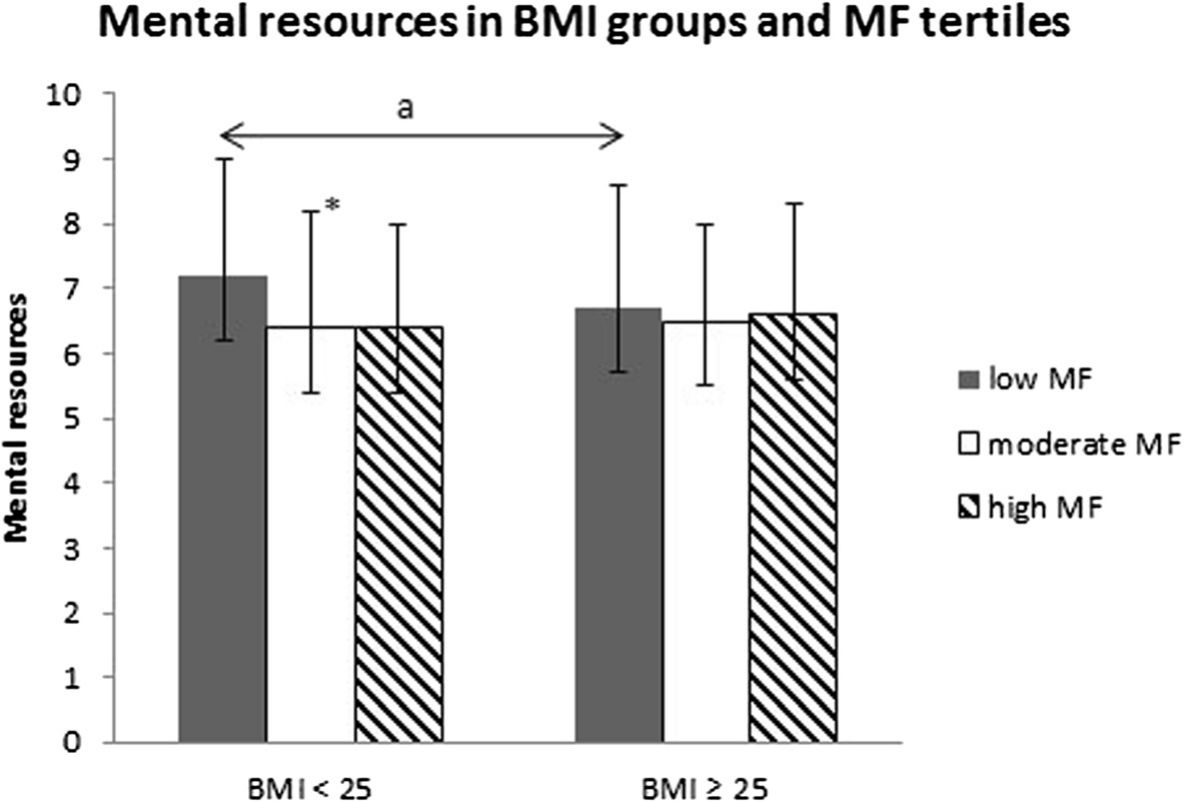
Mental resources according to Body Mass Index (BMI) groups and muscular fitness (MF) tertiles. Mean (±SD). The ANCOVA model contains age, tobacco and alcohol use, cardiorespiratory fitness (CRF) and leisure-time physical activity (LTPA) as covariates. Difference between the low and moderate/high subgroups within the BMI < 25 group: *0.01 < *p* ≤ 0.05

Overweight men with low MF (*n* = 130) reported 11 % (ES = 1.07, *p* = 0.003) less stress symptoms compared to their normal-weight (*n* = 105) counterparts with low MF. Overweight men with moderate (*n* = 91) MF experienced 6 % (ES = 0.47, *p* = 0.046) more stress symptoms compared to their normal-weight participants with moderate MF (*n* = 161). Overweight men with high MF (*n* = 74) experienced 7 % (ES = 0.61, *p* = 0.011) more stress symptoms compared to normal-weight men with high MF (*n* = 172) (Fig. 3). There were no significant differences in stress symptoms (p for trend, *p* = 0.30) or mental resources (p for trend, *p* = 0.92) within the overweight MF tertiles (low/moderate/high) (Figs. 3 and 4).

There were differences in mental resources between normal-weight and overweight men within MF tertiles. Normal-weight men with low MF reported 10 % (ES = 0.75, *p* = 0.034) lower mental resources compared to the normal-weight men with moderate MF and 11 % (ES = 0.83, *p* = 0.076, NS) lower mental resources compared to normal-weight men with high MF (p for trend was *p* < 0.001) (Fig. 4).

### Stress and mental resources according to BMI and LTPA

**Fig. 5:**
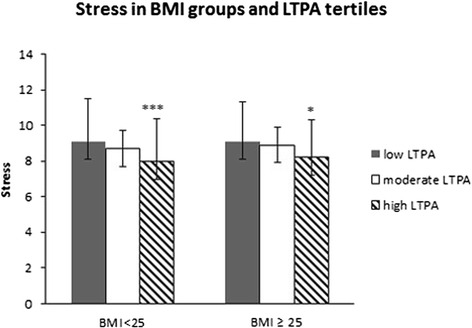
Stress symptoms according to Body Mass Index (BMI) groups and leisure time physical activity (LTPA) tertiles. Mean (±SD). The ANCOVA model contains age, tobacco and alcohol use and cardiorespiratory fitness (CRF) and muscle fitness (MF) as covariates. Differences between the low and moderate /high LTPA tertiles within the BMI < 25 group and BMI ≥ 25 group: *0.01 < *p* ≤ 0.05, **0.001 < *p* ≤ 0.01, ***p < 0.001

**Fig. 6:**
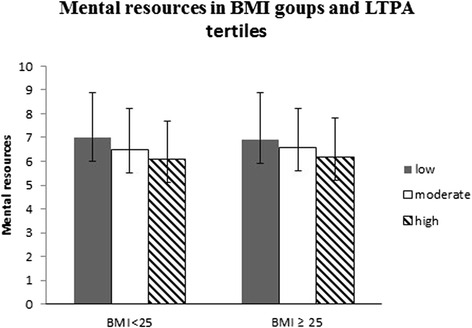
Mental resources according to Body Mass Index (BMI) groups and leisure-time physical activity (LTPA) tertiles. Mean (±SD). The ANCOVA model includes age, tobacco and alcohol use, cardiorespiratory fitness (CRF) and muscle fitness (MF) as covariates

Normal-weight men with low LTPA experienced 14 % (ES = 0.97, *p* < 0.0001) more stress symptoms compared to normal-weight men with high LTPA (p for trend, *p* < 0.001). The overweight men (*n* = 87, *p* < 0.001 for trend) with low LTPA reported 11 % (ES = 0.75, *p* = 0.047) more stress symptoms compared to the overweight men with high LTPA (p for trend, *p* = 0.009) (Fig. 5). There were no significant differences in mental resources between normal weight and overweight men (Fig. 6).

## Discussion

In an earlier study of the same study sample, it was found that higher levels of PF and PA were associated with lower stress and higher mental resources among young men [39]. The aim of the present study was to evaluate whether body weight would have an effect on these associations. We hypothesized on whether the association between mental health variables (stress and mental resources) would be different in normal-weight and overweight men. The present study showed that higher levels of cardiorespiratory and muscular fitness were associated with lower levels of stress and higher levels of mental resources among normal-weight men, but not in overweight men. Our hypothesis was correct; however, it is difficult to explain why this should be the case. Whether the “jolly fat” hypothesis stating that overweight people experience a lower rate of depressive symptoms than normal-weight people [10–13] would explain the result regarding stress symptoms is difficult to say and requires more evidence.

Our result shows that normal-weight men with low cardiorespiratory fitness experienced 13 % more stress symptoms than normal-weight men with higher CRF. This supports the evidence that CRF is associated with lower stress. In a meta-analysis of 73 studies by Jackson and Dishman [40], it was found that good cardiorespiratory fitness helps with recovery from stress. Physical fitness was likewise associated with psychological resources in a community health survey from New York, *n* = 1261 US citizens, both women and men [41]. On the contrary, Lindwall et al. [42] found no association between aerobic fitness (measured objectively using the Åstrand indirect test of maximal oxygen uptake (V0_2max_)) and mental health (measured with the Hospital Anxiety and Depression (HAD) questionnaire and the Shirom-Melamed Burnout Questionnaire (SMBQ)) in a sample of 177 voluntary subjects, both women and men. The score for the lowest CRF tertile was ml/kg/min, according to the range for CRF of the Finnish Defence Force. A secondary analysis was done, where the study group was divided in quintiles according to CRF. The lowest quintile was used as the low CRF group (<34.76 ml/kg/min, *n* = 153), the second and third lowest quintiles as the moderate CRF group (34.77–43.02 ml/kg/min, *n* = 317) and the two highest quintiles as the high CRF group (>43.03 ml/kg/min, *n* = 306). The same analyses (ANOVA and ANCOVA) were run for these new groups and the results were in line with the results of the original tertiles. However, one difference from the earlier results was observed: the mental resources became significant among normal-weight men within the low CRF group compared to the high group (*p* = 0.008).

In the present study, normal-weight men with low muscular fitness experienced 16 % more stress and 11 % lower mental resources than normal-weight men with high muscular fitness. There is, however, only limited evidence from previous studies concerning measured muscular fitness and stress [39] and, furthermore, we are unaware of previous studies examining body mass, measured muscular fitness and stress. Few studies have reported on the effects of strength training on mental health outcomes. Nevertheless, the small body of evidence is positive and concludes that strength training is associated with a reduction in anxiety symptoms among healthy adults [43].

Interestingly, overweight men with low physical fitness experienced somewhat less stress than normal-weight men with low physical fitness in both BMI x CRF and BMI x MF groups. This could be partly explained by the larger number of men with low physical fitness among the overweight men; 47 % (*n* = 160) of them had low CRF and 38 % (*n* = 130) low MF. Among normal-weight men, only 19 % (*n* = 94) had low CRF and 21 % (*n* = 105) low MF. The number of men with high physical fitness, in turn, was larger among normal-weight men. Of the normal-weight men, 43 % (*n* = 211) had high CRF and 35 % high MF; among the overweight men only 13 % (*n* = 44) had high CRF (*n* = 44) and 21 % (*n* = 74) high MF. According to some studies, overweight and obesity have a stronger positive correlation with poor levels of physical wellbeing than emotional wellbeing [6, 44]. We found negative correlations between BMI and both cardiorespiratory and muscular fitness. More studies are needed regarding the association between objectively measured physical fitness, overweight and stress in order to draw further conclusions.

In the present study, a high amount of moderate physical activity was associated with lower levels of stress in both normal and overweight men. In earlier studies, physically active persons seemed to be less reactive to psychological stressors [45, 46] and reported lower levels of stress [47, 48] and better mood states [47] than inactive persons. The association between PA and stress could be bidirectional [24]. The relationship between PA and mental health is likely to be complex and physical inactivity can be the cause and/or the consequence of poor mental health [25]. In this study, we used an analysis where stress symptoms and mental resources were selected as outcome variables. However, we did analyses in both directions of influence and these indicated that the results reported here were not identical when the influence was tested in the other direction. Nevertheless, we must keep in mind that this cross-sectional design cannot be used to draw conclusions regarding a causal relationship between the variables tested.

There is limited evidence of the relationship between stress, overweight PA and exercise, as only two studies among young people aged 8–24 years [2] have been conducted. To our knowledge, this was the first population sample to investigate the association between objectively measured physical fitness both CRF and MF together with self-reported LTPA overweight and stress in young men.

Our study sample is reasonably large and representative of Finnish young men. CRF and MF were objectively measured, but the cross-sectional design of the study reduces its power to establish causal relationships. The considerable heterogeneity in methods of defining and measuring stress makes it difficult to compare results with those of other studies. All these factors limit the generalization of the results of the study to other populations. However, despite the methodological diversity in subjectively measuring stress symptoms and mental resources, the results are mainly in accordance with earlier studies.

## Conclusion

In conclusion, higher cardiorespiratory and muscular fitness were associated with lower stress symptoms and higher mental resources in normal-weight men but not in overweight men. The current results underline how important a factor body weight might be when analysing associations between stress and physical fitness.

## Abbreviations

Abbreviations are defined in the text at first use, and hereby a list of abbreviations: ANCOVA, analysis of covariance; ANOVA, analysis of variance; BMI, body mass index; CRF, cardio respiratory fitness; HAD, hospital anxiety and depression questionnaire; HR, heart rate; ICC, intra class correlation; LTPA, leisure time physical activity; MF, muscular fitness; MFI, muscular fitness index; MR, mental resources; OSQ, occupational stress questionnaire; PA, physical activity; SIVAQ, single item question of leisure time vigorous physical activity; SMBQ, Shirom-Melamed Burnout Questionnaire; SPSS, statistical software package for social sciences; SS, stress symptoms
